# Exploring the mechanism of polymorphonuclear neutrophils against sepsis based on immune model

**DOI:** 10.1080/07853890.2026.2640268

**Published:** 2026-03-11

**Authors:** Chibo Liu, Yanqun Cai, Sihua Mou

**Affiliations:** Department of Clinical Laboratory Medicine, Taizhou Municipal Hospital (Taizhou University Affiliated Municipal Hospital), School of Medicine, Taizhou University, Taizhou Key Laboratory of Infection and Tumor Immunology, Taizhou, Zhejiang, China

**Keywords:** Sepsis, polymorphonuclear neutrophils, immune model

## Abstract

**Background:**

Sepsis is a life-threatening organ dysfunction caused by a dysregulated host response to infection and remains a major global health challenge. Polymorphonuclear neutrophils (PMNs), as major effectors of innate immunity, are essential for antimicrobial defense but can also contribute to immune dysregulation, tissue injury, and organ failure during sepsis.

**Methods:**

We conducted a narrative review of the literature by searching PubMed and Web of Science from database inception to November 2025. Search terms included sepsis, septic shock, neutrophils, polymorphonuclear neutrophils, PMNs, immunology, immune models, diagnosis, biomarkers, and treatment. Peer-reviewed English-language studies and reviews focusing on neutrophil biology, immune mechanisms, diagnostic applications, and therapeutic strategies in sepsis were included.

**Results:**

Current evidence shows that PMNs play a dual role in sepsis. On the one hand, they mediate pathogen clearance through chemotaxis, phagocytosis, reactive oxygen species production, degranulation, and neutrophil extracellular trap formation. On the other hand, excessive or dysregulated PMN activation amplifies inflammation, disrupts endothelial and microvascular integrity, alters cellular metabolism, and promotes organ dysfunction. Sepsis is also associated with marked neutrophil phenotypic and functional changes, including altered surface marker expression, impaired migration and phagocytosis, glycolytic reprogramming, and abnormal intercellular signaling. Emerging biomarkers, immune-related prognostic models, and artificial intelligence-assisted approaches may improve risk stratification and individualized management.

**Conclusions:**

PMNs are central to the immunopathogenesis of sepsis and represent promising biomarkers and therapeutic targets. Further studies on neutrophil heterogeneity, metabolic adaptation, and immune interactions may support the development of more precise diagnostic and immunomodulatory strategies.

## 1. Introduction

Sepsis is a life-threatening organ dysfunction caused by a dysregulated host response to infection, involving complex pathophysiological mechanisms [1]. A hallmark of sepsis is excessive inflammation, characterized by massive release of cytokines such as tumour necrosis factor-α (TNF-α), interleukin-1β (IL-1β) and IL-6. This cascade triggers systemic inflammatory response syndrome (SIRS), leading to tissue and organ damage [2]. Concurrently, sepsis induces circulatory and microvascular alterations, including vascular endothelial dysfunction, increased permeability, and extravasation of fluid and proteins that cause tissue oedema [3]. Additional contributors to sepsis pathogenesis include impaired nitric oxide (NO) synthesis, dysregulated calcium homoeostasis, abnormal cardiac autophagy, autonomic nervous system dysfunction and metabolic reprogramming [1,4].

The global incidence of sepsis is rising, posing significant public health challenges [5]. It exhibits distinct epidemiological patterns, with higher prevalence among elderly individuals, males and specific racial/ethnic groups [6]. Pathogen distribution varies; Gram-positive bacteria remain predominant, though fungal pathogens are rapidly increasing [7]. Common infection sites include the respiratory tract and bloodstream [8,9].

Polymorphonuclear neutrophils (PMNs), key effector cells of innate immunity, play critical roles in host defense [10,11]. Notably, they exhibit selective cytotoxicity against four human cancer cell lines while sparing normal epithelial cells [10]. During bacterial infections, PMNs detect pathogen-associated molecular patterns (PAMPs; e.g. uncapped non-polyadenylated prokaryotic mRNA) to sense bacterial viability. This triggers activation events including increased forward scatter, CD11b surface expression, chemotaxis, reactive oxygen species (ROS) generation and neutrophil extracellular trap (NET) formation [12,13]. PMNs-derived exosomes further modulate immunity by activating macrophage signalling pathways (e.g. Mer tyrosine kinase [MerTK], Ca^2+^ flux, and rapid TGF-β1 release) [14,15]. PMNs function and phenotype are context-dependent: in primary myelofibrosis, expanded polymorphonuclear myeloid-derived suppressor cells (PMNs-MDSCs) contribute to pathogenesis [16,17]. PMNs respond more robustly to live bacteria than other states, with functions regulated by factors like NO (affecting chemotaxis, ROS production, and NETosis) [18,19].

Mitochondrial dysfunction is central to sepsis pathophysiology [1,20]. Structural abnormalities, oxidative stress, aberrant mitochondrial permeability transition pore (mPTP) opening, uncoupling and impaired quality control disrupt homeostasis, promoting sepsis progression [21]. For instance, mitochondrial impairment underlies sepsis-induced myocardial dysfunction (SIMD), compromising cardiac contractility and relaxation [1,22]. Endothelial microvesicles further exacerbate sepsis by inducing pulmonary vascular leakage and injury *via* miR-23b-mediated targeting of tight junction protein ZO-1, highlighting the disease’s mechanistic complexity [23,24].

In sepsis patients, PMNs undergo significant alterations. Surface marker CD64 is upregulated and serves as a sensitive/specific biomarker for distinguishing sepsis from postoperative SIRS [25]. Metabolically, PMNs exhibit altered glycolysis. Metabolomic analysis of PMNs from 14 septic patients, 26 acute appendicitis patients, and 19 healthy volunteers revealed profound Warburg effect modifications. The PI3K/Akt/HIF-1α pathway regulates this glycolytic shift, impairing PMNs chemotaxis and phagocytosis [26].

This review examines the immunology of neutrophils in sepsis, detailing PMNs biology, sepsis pathophysiology and immune system interactions to provide novel perspectives.

## 2. Methods

This review is based on a narrative literature search. We searched the PubMed and Web of Science databases, covering the period from database inception to November 2025, using combinations of the following keywords and MeSH terms: ‘sepsis’ or ‘septic shock’ and ‘neutrophil*’, ‘polymorphonuclear neutrophil*’, ‘PMNs’, in combination with terms such as ‘immunology’, ‘immune model*’, ‘animal model*’, ‘*in vitro*’, ‘*ex vivo*’, ‘diagnosis’, ‘biomarker’, ‘treatment’ and ‘immunotherapy’. In addition, we screened the reference lists of key original studies and recent related reviews to identify additional relevant publications.

The inclusion criteria were as follows: peer-reviewed original research articles or reviews published in English that primarily addressed neutrophil (polymorphonuclear neutrophils, PMNs) biology, functional characteristics, immune/animal models, diagnostic strategies or therapeutic approaches in the context of sepsis or septic shock. Conference abstracts, non–peer-reviewed publications, editorials lacking primary data, and articles whose main focus was not sepsis-related neutrophil immunology were not systematically considered. Given that this work is a narrative rather than a strictly systematic review, we did not perform a formal systematic screening process (such as PRISMA) or quantitative risk-of-bias assessment.

## 3. Results and discussion

### 3.1. Mechanisms of PMNs in sepsis

#### 3.1.1. Role of PMNs in sepsis pathogenesis

PMNs exhibit notable heterogeneity across different populations [27]. Age significantly influences PMNs function and behaviour in certain diseases [28]. For instance, in mycoplasma pneumoniae pneumonia (MPP), advanced age is a prognostic factor associated with higher PMNs counts, enhanced functional activity, and distinct profiles in neutrophil apoptosis, NADPH metabolism, mitochondrial function, and oxidative stress [29].

Disease states also drive PMNs variations. Cancer patients demonstrate altered PMNs phenotypes and functions compared to healthy individuals. Within tumour microenvironments, which exemplified in pancreatic cancer, PMNs infiltration inversely correlates with E-cadherin expression. PMNs promote tumour cell migration and invasive growth through elastase-mediated degradation of E-cadherin [30]. Similarly, in primary myelofibrosis, circulating PMNs-myeloid-derived suppressor cells (PMNs-MDSCs) contribute to disease pathogenesis and phenotypic heterogeneity [31]. Furthermore, during infectious diseases like Mycobacterium tuberculosis infection, pathogen-specific responses occur, including PMNs-derived exosomes that modulate immune responses. PMNs activation patterns vary considerably depending on pathogen type and infection mechanisms [32].

PMNs play a crucial and complex role in the pathogenesis of sepsis (Table 1). For instance, in a rat caecal ligation and puncture (CLP) model of sepsis, before ventilation, CLP rats had higher levels of alveolar neutrophils and interleukin-1β, and after 60 min of ventilation, they showed worse lung injury, indicating that PMNs are involved in the early inflammatory response and the subsequent development of tissue damage in sepsis [10,11,33].

**Table 1.** Role of PMNs in sepsis pathogenesis.

Pathogenic Mechanism	Molecular/Cellular Basis	Functional Consequences	References
Hyperactivation and Excessive Inflammation	TLR/NF-κB activation by PAMPs/DAMPs → ROS, cytokines (IL-1β, TNF-α). Complement (C5a)-driven chemotaxis.	Cytokine storm → endothelial damage. Systemic inflammation (SIRS).	[33,44]
Dysregulated NETosis	Histone citrullination (PAD4) → chromatin decondensation. MPO/NE release with DNA extrusion.	Microthrombosis (platelet/NET complexes). Direct tissue injury (protease/oxidative stress).	[12,14,17]
Prolonged Survival and Delayed Apoptosis	G-CSF, GM-CSF, LPS → inhibit pro-apoptotic signals (Bax/Bak). Upregulation of anti-apoptotic Mcl-1.	Accumulation in tissues → sustained inflammation. Necrotic cell death → DAMPs release.	[39,41]
Immunosuppressive Functions	Arginase-1 secretion → T-cell suppression. PD-L1 expression → lymphocyte exhaustion. IL-10 release → anti-inflammatory shift.	Impaired bacterial clearance. Secondary infections (e.g., pneumonia).	[42,45,47]
Endothelial Injury and Vascular Leak	β2-integrin (CD11b/CD18) binding to ICAM-1. NE/MMP-9 degradation of endothelial junctions.	Capillary leakage → hypovolemia. Leukocyte extravasation → tissue edema.	[31,50]
Mitochondrial Dysfunction	ROS-induced mtDNA damage → impaired ATP synthesis. Reduced oxidative burst → ineffective bacterial killing.	Energy failure in immune cells. Paradoxical susceptibility to infections.	[39,50]

**Figure 1.**
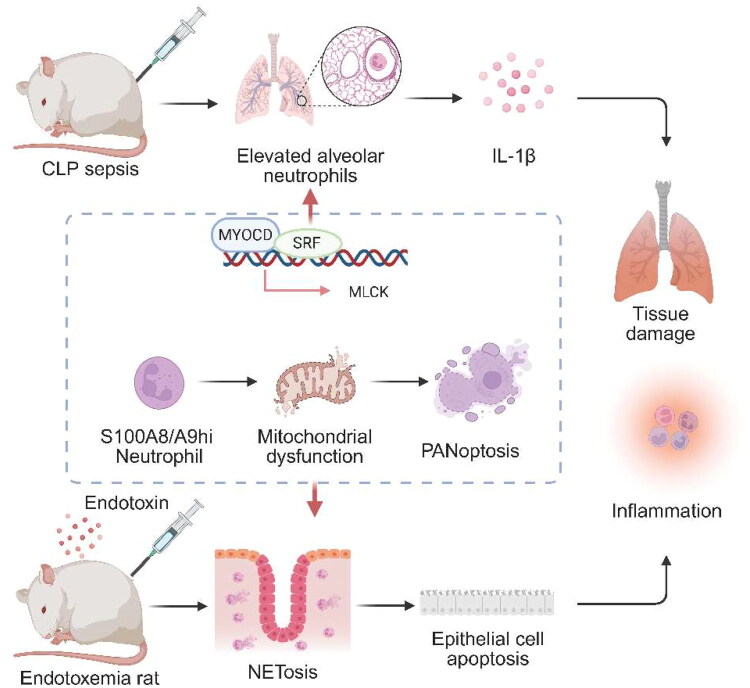
PMNs programmes driving sepsis pathogenesis. In CLP rats, alveolar PMNs and IL-1β levels are elevated, indicating that PMNs-driven early inflammatory responses contribute to subsequent tissue injury. NETs also participate in pathogenesis: in endotoxemic rats, PMNs infiltrate the intestinal mucosa and are activated to form NETs; degrading NETs attenuates the acute systemic inflammatory response and reduces intestinal epithelial cell apoptosis. Mechanistically, MYOCD and SRF cooperatively upregulate MLCK transcription, enhancing endothelial contraction, increasing permeability, and facilitating PMNs transendothelial migration. In parallel, S100A8/A9hi PMNs induce complex I deficiency–driven endothelial mitochondrial dysfunction and PANoptosis, leading to endothelial cell death and barrier disruption that further amplifies inflammation and organ damage. PMNs, polymorphonuclear neutrophils; IL-1β, interleukin-1β; NETs, neutrophil extracellular traps; MYOCD, myocardin; SRF, serum response factor; MLCK, myosin light chain kinase; PANoptosis, pyroptosis, apoptosis and necroptosis.

Neutrophil extracellular traps (NETs), which are expelled from activated neutrophils, also contribute to the pathogenesis (Figure 1). NETs were first described in 2004 as extracellular chromatin fibres decorated with histones and granular proteins that can trap and kill microbes [34]. This process of NET formation, later termed ‘NETosis’, refers to a distinct neutrophil death or activation programme. It is characterized by chromatin decondensation, nuclear envelope breakdown, and extrusion of NET structures. Mechanistically, NETosis differs from canonical apoptosis and necrosis [35]. In endotoxemic rats, a large number of neutrophils infiltrated and were activated to release NETs in the intestine and the disruption of NETs reduced the acute systemic inflammatory response, apoptosis of intestinal epithelial cells and alleviated histologic pathogenesis, suggesting that the release of NETs may contribute to the intestinal damage during sepsis [12,15]. Moreover, in a murine model of lipopolysaccharide (LPS)-induced sepsis, the role of NETs in sepsis, particularly the balance between their antimicrobial and cytotoxic actions, remains unclear [17]. Neutrophils from peptidylarginine deiminase 4-(PAD4(-/-)) deficient mice, which lack the enzyme allowing for chromatin decondensation and NET formation, were evaluated. It was found that prevention of NET formation might not have devastating consequences in sepsis, as PAD4(-/-) mice did not fare worse than wild-type mice in a mild and severe polymicrobial sepsis model [14,36]. However, these findings are derived exclusively from small-mammal, predominantly hypodynamic models and cannot be directly extrapolated to hyperdynamic septic shock in larger species or humans. Hyperdynamic porcine and ovine sepsis models more closely reproduce human hemodynamics, but NET-targeted interventions have not yet been systematically evaluated in these settings; only limited work in lipopolysaccharide-induced sepsis in aged mini pigs has shown that hydrogen gas inhalation can suppress NET formation *in vivo* without providing definitive survival data [37,38].

Beyond hyperactivation and NET release, sepsis profoundly alters PMNs lifespan. During early and established sepsis, circulating neutrophils exhibit markedly delayed apoptosis, characterized by preserved mitochondrial membrane potential, reduced caspase-9 and caspase-3 activation, and prolonged survival compared with neutrophils from healthy controls [39,40]. Pro-survival signals such as bacterial products (e.g. LPS) and cytokines including granulocyte colony-stimulating factor (G-CSF) and granulocyte–macrophage colony-stimulating factor (GM-CSF) activate NF-κB and PI3K–Akt pathways. These pathways upregulate anti-apoptotic Bcl-2 family members such as myeloid cell leukemia-1 (Mcl-1) and suppress pro-apoptotic mediators. Together, these effects extend PMNs lifespan and sustain inflammatory tissue damage in sepsis [41]. As summarized in Table 1, this prolonged survival and delayed apoptosis of PMNs represents a key mechanism linking the initial inflammatory burst to persistent organ injury.

With disease progression, PMNs also acquire potent immunosuppressive functions. A proportion of circulating and low-density PMNs in septic shock patients displays myeloid-derived suppressor cell–like properties, with high expression of arginase-1 that metabolizes L-arginine and thereby constrains T-cell proliferation and CD3ζ-chain expression; depletion or inhibition of these PMNs-MDSCs restores T-cell function *ex vivo* [42,43]. Transcriptomic and functional studies further confirm that neutrophil arginase-1 signalling suppresses polyfunctional effector CD8^+^ T-cell responses, whereas pharmacologic blockade of arginase-1 rescues interferon-γ and granzyme B production [43]. In parallel, neutrophils in sepsis upregulate the immune checkpoint ligand programmed death-ligand 1 (PD-L1) under inflammatory and metabolic stress, which contributes to T-cell exhaustion, reduced antimicrobial effector functions, and, in experimental models, worsened lung injury and mortality [44–46]. Moreover, neutrophils become an important source of the anti-inflammatory cytokine interleukin-10 (IL-10) during polymicrobial sepsis; neutrophil-derived IL-10 suppresses peritoneal inflammatory monocytes and promotes an immunosuppressed milieu that predisposes patients to secondary infections [11,47]. Collectively, these immunosuppressive PMNs phenotypes help explain the transition from early hyperinflammation to late immune paralysis in sepsis (Table 1).

Furthermore, PMNs play a critical role in sepsis-associated acute respiratory distress syndrome (ARDS). RNA sequencing in murine models reveals myosin light chain kinase (MLCK) as the most significantly differentially expressed gene in PMNs from septic ARDS mice versus controls. Mechanistically, myocardin (MYOCD) and serum response factor (SRF) promote MLCK transcription [33]. Knockdown of MLCK, MYOCD, or SRF ameliorates pulmonary dysfunction and edema, implicating this signaling axis in PMNs-mediated lung injury during sepsis [48,49]. Concurrently, S100A8/A9hi neutrophils in septic patients induce endothelial mitochondrial dysfunction and PANoptosis through mitochondrial complex I deficiency. Notably, elevated S100A8 levels constitute an independent prognostic risk factor for adverse outcomes in these patients [50].

#### 3.1.2. Interaction between PMNs and other immune cells in sepsis

**Figure 2.**
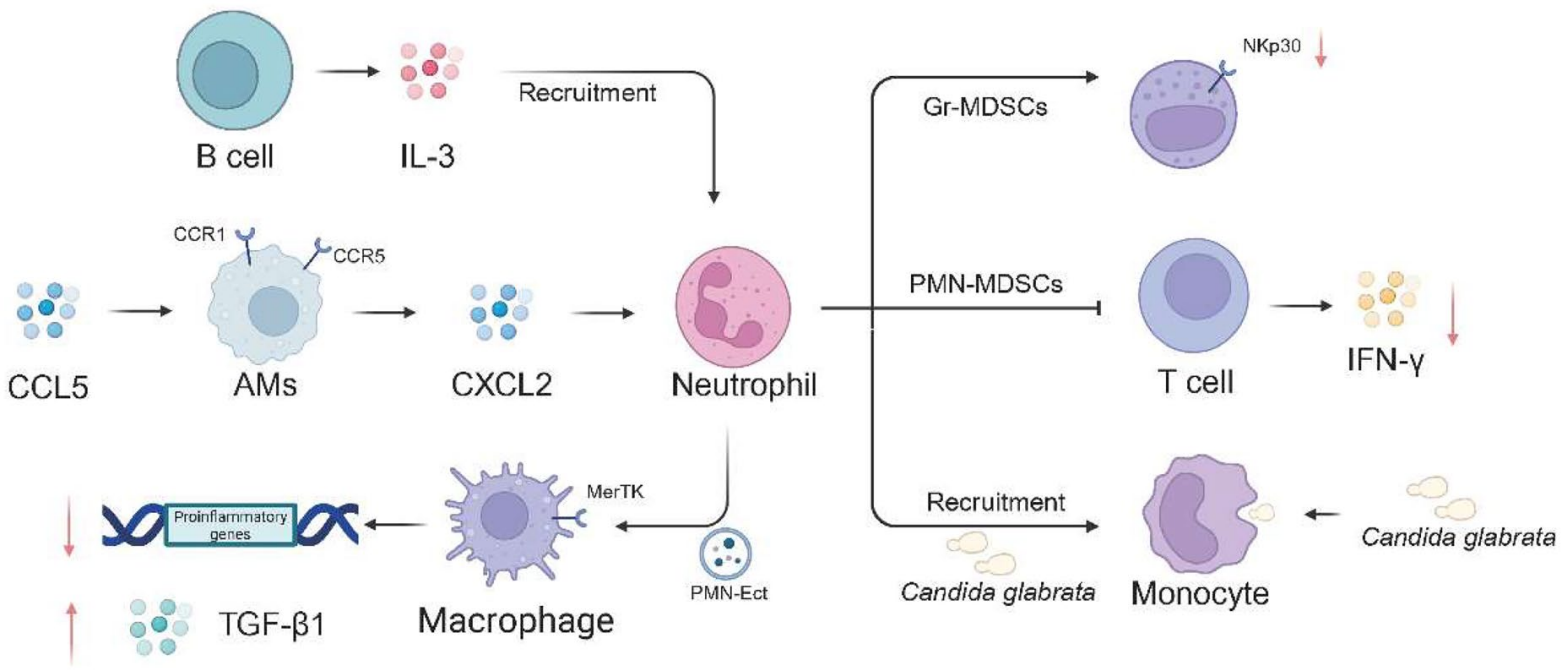
Immunomodulatory roles of PMNs and derived mediators in sepsis. In the CLP sepsis model, plasma levels of CCL5 are elevated; alveolar macrophages highly express CCR1/CCR5, and CCL5 stimulates isolated alveolar macrophages to secrete CXCL2, suggesting that platelets regulate neutrophil recruitment by releasing CCL5 to activate alveolar macrophages. Innate immune responses activate B cells to produce IL-3, which induces emergency myelopoiesis of neutrophils and fuels the cytokine storm. In the interaction between neutrophils and monocytes, the supernatant from neutrophils co-incubated with *Candida glabrata* attracts monocytes and enhances their phagocytosis of the fungus. PMNs-derived extracellular vesicles (PMNs-Ect) act through the phosphatidylserine (PS)–MerTK axis to inhibit p65 phosphorylation and nuclear translocation, thereby suppressing NF-κB signalling and proinflammatory gene transcription; simultaneously, they induce rapid Ca^2+^ flux and trigger TGF-β1 release from cytosolic stores, collectively reprogramming the immunoregulatory functions of macrophages. During sepsis, PMNs-MDSCs inhibit T-cell proliferation and IFN-γ production, thereby enhancing immunosuppression; additionally, Gr-MDSCs downregulate NKp30 expression, weakening NK cell antifungal activity. CLP, caecal ligation and puncture; CCL5, C-C motif chemokine ligand 5; CCR, C-C chemokine receptor; CXCL2, C-X-C motif chemokine ligand 2; IL-3, interleukin-3; PMNs, polymorphonuclear neutrophil; PMNs-Ect, PMNs-derived extracellular vesicles; PS, phosphatidylserines; MerTK, MER proto-oncogene tyrosine kinase; NF-κB, nuclear factor kappa B; TGF-β1, transforming growth factor beta 1; PMNs-MDSC, polymorphonuclear myeloid-derived suppressor cell; Gr-MDSC, granulocytic myeloid-derived suppressor cell; NK, natural killer; IFN-γ, interferon-gamma.

The interaction between PMNs and other immune cells is a key aspect of the immune response during sepsis (Table 2). Platelets, for example, play an important role in regulating neutrophil recruitment in septic lung injury [19]. In a study where abdominal sepsis was induced by CLP in C57BL/6 mice, it was found that CLP increased plasma levels of CCL5, a chemokine. Platelet depletion and treatment with the Rac1 inhibitor NSC23766 markedly reduced CCL5 in the plasma of septic mice [48]. Immunoneutralization of CCL5 decreased CLP-induced neutrophil infiltration, oedema formation and tissue injury in the lung. Isolated alveolar macrophages expressed significant levels of the CCL5 receptors CCR1 and CCR5, and CCL5 triggered significant secretion of CXCL2 from isolated alveolar macrophages, suggesting that platelets can regulate neutrophil recruitment *via* activation of alveolar macrophages through the secretion of CCL5 (Figure 2) [16,51].

**Table 2.** Interaction between PMNs and immune cells in sepsis.

Interacting Cell Type	Key Interaction Mechanisms	Functional Impact in Sepsis	References
Monocytes/Macrophages	PMNs → MΦ: NETs induce inflammasome activation (e.g., IL-1β). MΦ → PMNs: TNF-α, IL-8, G-CSF enhance PMNs recruitment & survival.	Amplifies pro-inflammatory response. Drives early cytokine storm.	[4,14,52]
Lymphocytes (T cells)	PMNs suppress T cells: ROS/arginase-1 deplete arginine → inhibit T-cell proliferation. Th17/PMN crosstalk: IL-17 promotes PMNs recruitment.	T-cell exhaustion/apoptosis → immunosuppression. Th17 response fuels inflammation.	[42,43]
Dendritic Cells (DCs)	NETs impair DCs: Disrupt antigen presentation → reduce T-cell activation. PMNs proteases (e.g., elastase) degrade DC cytokines.	Compromised adaptive immunity initiation.	[63]
Mast Cells	PMNs proteases (cathepsin G) activate mast cells → histamine release. Mast cell TNF-α recruits PMNs.	Synergistic vascular leakage & hypotension.	[63,64]
Platelets	P-selectin/PSGL-1 binding → PMNs-platelet aggregates. Mutual activation: Platelets enhance NETosis; NETs activate platelets.	Microthrombosis & endothelial injury. Amplifies coagulopathy.	[31]
Myeloid-Derived Suppressor Cells (MDSCs)	PMNs induce MDSC expansion via IL-10, S100A8/9. MDSCs suppress PMNs apoptosis.	Enhanced immunosuppression & PMNs persistence in tissues.	[16,42,43]
Complement System	C5a → PMNs activation: Chemotaxis, ROS, NETosis. PMNs proteases cleave complement factors (e.g., C3, C5).	Uncontrolled inflammation & tissue injury.	[60,63]
Endothelial Cells	PMNs adhesion (ICAM-1/β2-integrins) → transmigration. NETs induce endothelial death → barrier disruption.	Vascular leak, edema, thrombosis.	[33,50]

In addition, the cytokine interleukin-3 (IL-3) potentiates inflammation in sepsis. Using a mouse model of abdominal sepsis, it was shown that innate response activator B cells produce IL-3, which induces myelopoiesis of Ly-6C(high) monocytes and neutrophils and fuels a cytokine storm [52]. IL-3 deficiency protects mice against sepsis, indicating the complex interplay between B cells, PMNs, and monocytes in the context of sepsis [18]. Moreover, in the interaction between neutrophils and monocytes, supernatants from co-incubations of neutrophils with *Candida glabrata* primarily attracted monocytes and increased their phagocytosis of *C. glabrata*. In contrast, PMNs activation by *Candida albicans* resulted in the recruitment of more neutrophils. These findings demonstrate differential targeting of immune cell populations by different Candida species [1,4].

PMNs-derived exosomes (PMNs-Ect) regulate macrophage function through phosphatidylserine (PS)-mediated MerTK receptor activation. This activation suppresses NF-κB signalling by inhibiting p65 phosphorylation and nuclear translocation. As a result, pro-inflammatory gene transcription is reduced. Concurrently, PMNs-Ect induces rapid Ca^2+^ flux and triggers TGF-β1 release from cytoplasmic stores, collectively reprogramming macrophage immunoregulatory function [48].

Although PMNs primarily mediate innate immunity while T lymphocytes dominate viral/tumour defense, emerging evidence reveals significant cross-talk. In pathological contexts including acute/chronic inflammation, anti-tumour immunity and pregnancy maintenance, these cells interact through cytokine networks to co-regulate immune responses [51,53]. Sepsis amplifies this interaction through polymorphonuclear myeloid-derived suppressor cells (PMNs-MDSCs), which suppress T-cell proliferation and IFN-γ production, exacerbating immunosuppression [54]. PMNs also modulate natural killer (NK) cell function: during Aspergillus infections, granulocytic MDSCs (Gr-MDSCs) impair antifungal immunity by downregulating NKp30 expression and cytotoxicity against A. fumigatus [55].

PMNs initiate anti-septic responses *via* pattern recognition receptor (PRR) detection of viability-associated pathogen-associated molecular patterns (PAMPs), such as uncapped non-polyadenylated prokaryotic mRNA. This recognition triggers bactericidal programmes including chemotaxis, ROS generation and NETosis [56]. Pathogen viability dictates response intensity – live *E. coli* potently induces forward scatter elevation, CD11b upregulation, enhanced chemotaxis, ROS production and NET formation, while heat-killed bacteria fail to elicit comparable activation [56,57].

Over the years, research on PMNs in sepsis has evolved significantly. Early studies began to recognize the involvement of PMNs in the host response to sepsis [58]. For example, in a murine model of sepsis, it was demonstrated that neutrophils are significant producers of IL-10 at the site of infection during sepsis, highlighting their role in the immunoregulatory cytokine network [59,60].

More recently, advanced techniques have been used to further understand the role of PMNs. RNA sequencing has been employed to identify key molecular mechanisms involved in the hyperactivation of PMNs during sepsis-related ARDS [61]. In a mouse model of sepsis-related ARDS generated by lipopolysaccharide (LPS) injection, myosin light chain kinase (MLCK) was identified as the most significant differentially expressed gene between PMNs isolated from model and control mice [62]. Myocardin (MYOCD) and serum response factor (SRF) were found to promote the transcription of MLCK by binding to its promoter, and knockdown of MLCK, MYOCD, or SRF ameliorated dysfunction and oedema in the lungs of LPS-treated mice [63,64].

Furthermore, the role of PMNs-derived exosomes in sepsis has emerged as an important area of research. It has been shown that PMNs-derived exosomes promote the formation of neutrophil extracellular traps (NETs) and induce multiple organ dysfunction during sepsis [65]. Administration of LPS-stimulated PMNs-derived exosomes in mice significantly enhanced NET formation, resulting in multi-organ inflammation and tissue injury. *In vitro* coculture experiments also demonstrated that exosomes from LPS-stimulated PMNs promote ROS-dependent NET formation, and proteomic analysis revealed enrichment of matrix metalloproteinase 9 (MMP9) expression in these exosomes, with exosomal MMP9 inducing NET formation through the p38 MAPK pathway [66,67].

### 3.2. Immune model frameworks in sepsis

#### 3.2.1. Development of immune models for sepsis

The development of immune models for sepsis has been crucial in understanding the complex pathophysiology of the disease. Animal models, such as the caecal ligation and puncture (CLP) model in mice, have been widely used [68]. The CLP model in rodents closely mimics human abdominal infection-induced sepsis, thereby serving as a critical platform for investigating sepsis pathophysiology and evaluating novel therapeutic approaches [69]. CLP studies demonstrate that during sepsis development, host immune cells, including but not limited to neutrophils and macrophages, undergo significant alterations in both quantity and function, accompanied by dysregulated inflammatory mediator expression [70,71]. In addition to rodent CLP models, endotoxemia induced by intravenous LPS infusion in large animals such as horses has been used as an experimental platform to explore immunomodulatory therapies. For example, a randomized placebo-controlled trial evaluated the effect of intravenous administration of peripheral blood-derived mesenchymal stromal cells (PB-MSCs) in LPS-challenged horses. At the dose tested, PB-MSC infusion did not significantly alter clinical signs, clinicopathological variables, or inflammatory cytokine gene expression at any time point [72,73]. These data primarily inform the feasibility and short-term safety of MSC-based interventions in a large-animal endotoxemia model. However, given the marked inter-species differences in LPS susceptibility and cardiovascular responses, and the fact that humans are among the most LPS-sensitive species, no direct conclusions can be extrapolated from the equine LPS model to human sepsis.

*In vitro* immune cell models provide critical insights into sepsis mechanisms, enabling investigation of signal transduction pathways and inflammatory mediator release. Studies using human peripheral blood monocytes reveal that PD-L1 (programmed death-ligand 1) expression in septic patients correlates with endotoxin tolerance following LPS stimulation, where PD-L1 mediates T-cell proliferation suppression [22]. Complementing experimental approaches, computational modelling facilitates sepsis progression prediction and therapeutic efficacy assessment, generating theoretical frameworks to guide clinical management [74].

Viral-based models have also been explored. A virotherapy using the nonpropagative modified vaccinia virus Ankara (MVA) to deliver IL-7 was developed. The rMVA-human IL-7 (hIL-7)-Fc encoding the hIL-7 fused to the human IgG2-Fc was engineered and shown to express a dimeric, glycosylated, and biologically active cytokine [15]. Following a single i.v. injection in naive mice, it increased the number of total and activated B, T, and NK cells, as well as myeloid subpopulations. The MVA-hIL-7-Fc also conferred a significant survival advantage in the CLP and Candida albicans sepsis models, highlighting the potential of such viral-based immune models in studying sepsis immunotherapy [75].

#### 3.2.2. PMNs-focused mechanistic and therapeutic insights in sepsis

PMNs harbour multiple promising therapeutic targets for sepsis management. Notably, strategic modulation of PMNs metabolic pathways represents an emerging therapeutic frontier [76]. Research has demonstrated that during sepsis, PMNs undergo significant alterations in glucose metabolism—specifically, a pronounced shift toward aerobic glycolysis (Warburg effect) [26]. Critically, pharmacological or genetic targeting of key glycolytic enzymes such as lactate dehydrogenase A (LDHA) can directly regulate neutrophil effector functions, including chemotaxis, phagocytosis, and NETosis. This metabolic reprogramming approach offers novel mechanistic insights and potential clinical interventions for sepsis [26].

Furthermore, therapeutic strategies targeting PMNs surface receptors and downstream signaling cascades show considerable promise. Selective blockade of pattern recognition receptors (such as TLR4) or cytokine receptors (such as IL-1R) could attenuate pathological hyperinflammation by reducing excessive pro-inflammatory mediator release [77]. Complementarily, interventions modulating intercellular crosstalk between PMNs and other immune cells, particularly through regulation of soluble mediators like chemokines (CXCL8), complement components (C5a) and alarmins (S100A8/A9), may restore immunological balance [78,79]. Such approaches could simultaneously mitigate early hyperinflammation and prevent late-phase immunosuppression in sepsis pathogenesis.

#### 3.2.3. Limitations of current immune models in sepsis research

Current immunological models for sepsis research exhibit inherent limitations. While animal models can recapitulate certain pathophysiological aspects of sepsis, significant interspecies differences persist between these models and human sepsis. For instance, although the CLP model in mice successfully induces systemic inflammatory response syndrome (SIRS), fundamental distinctions in immune architecture and physiological characteristics between murine and human systems may compromise translational relevance [80]. These differences include variations in leukocyte subpopulation distributions, cytokine kinetics and organ-specific responses, potentially introducing extrapolation bias when translating preclinical findings to clinical contexts [81].

*In vitro* cell models present inherent limitations due to the fundamental gap between artificial culture conditions and the physiological microenvironment *in vivo*. These systems often fail to recapitulate the dynamic reciprocity of intercellular crosstalk and signal transduction networks observed in living organisms [82]. Furthermore, while mathematical modeling and computational simulations offer valuable predictive frameworks for sepsis progression, their accuracy remains contingent upon both the quality of input data (requiring extensive experimental validation) and the biological plausibility of underlying assumptions [83]. Practical implementation may be compromised by parameter uncertainty, unmodeled biological variables, and evolving disease dynamics [84]. Consequently, concerted refinement of immunological models is imperative to achieve higher-fidelity recapitulation of human sepsis pathophysiology.

In general, animal models may not fully recapitulate the human septic milieu. There are significant differences in the immune response between species, and the complex interactions between the host, pathogens, and the immune system in humans may not be accurately replicated in animal models, limiting the translation of findings from these models to clinical practice [85]. Moreover, even within humans, there is substantial immunological heterogeneity between men and women: females generally mount stronger innate and adaptive immune responses than males and exhibit distinct patterns of sepsis incidence, cardiovascular responses, and outcomes, with male sex often associated with a higher risk of developing sepsis and worse prognosis. These sex-related differences further complicate the extrapolation of preclinical data to a heterogeneous patient population [86,87].

### 3.3. Diagnostic and treatment strategies for PMNs and sepsis

#### 3.3.1. Diagnostic techniques for PMNs and sepsis

Assessment of PMNs functionality provides critical insights for evaluating sepsis severity and prognosis. Standardized assays quantify key effector functions including phagocytic capacity, oxidative burst activity and chemotaxis. Phagocytosis is typically measured using fluorescence-labelled bacteria or particles, while chemiluminescence or flow cytometry detects stimulus-induced reactive oxygen species (ROS) production to assess oxidative burst [88].

Additional functional metrics include secretion profiling of enzymes (myeloperoxidase [MPO], elastase [EL]) and cytokines (IL-8), which reflect immunomodulatory activity. In sepsis patients, dysregulated release patterns of these mediators correlate with disease severity [89]. Surface marker phenotyping (CD11b, CD64, CD62L) further delineates PMNs activation states and functional alterations [90].

Identification of robust sepsis biomarkers remains essential for early diagnosis, disease monitoring, and prognostic stratification. Well-established markers include procalcitonin (PCT), C-reactive protein (CRP), and interleukin-6 (IL-6). PCT demonstrates high specificity for bacterial sepsis, with circulating levels correlating with infection severity to guide antibiotic stewardship [91]. CRP, though rapidly elevated during sepsis, exhibits modest specificity due to concomitant increases in non-infectious inflammatory conditions. IL-6 serves as an early-rising biomarker whose kinetics reflect disease trajectory and mortality risk [92]. Emerging markers like heparin-binding protein (HBP) and kidney injury molecule-1 (KIM-1) show promising diagnostic and prognostic utility in validation cohorts, suggesting potential diagnostic and prognostic value; however, their exact pathophysiological roles and clinical utility remain to be fully elucidated [93,94].

Immunodiagnostic models augment conventional sepsis diagnostics. Flow cytometric profiling of immune cell phenotypes integrated with machine learning algorithms enables high-accuracy discrimination between septic patients and healthy controls [95]. Transcriptomic approaches identify sepsis-specific gene signatures in immune cells, facilitating early detection through predictive modelling [96]. *In vivo* and *in vitro* models provide essential platforms for validating novel biomarkers and diagnostic strategies prior to clinical implementation [97].

#### 3.3.2. Treatment strategies for PMNs and sepsis

**Figure 3.**
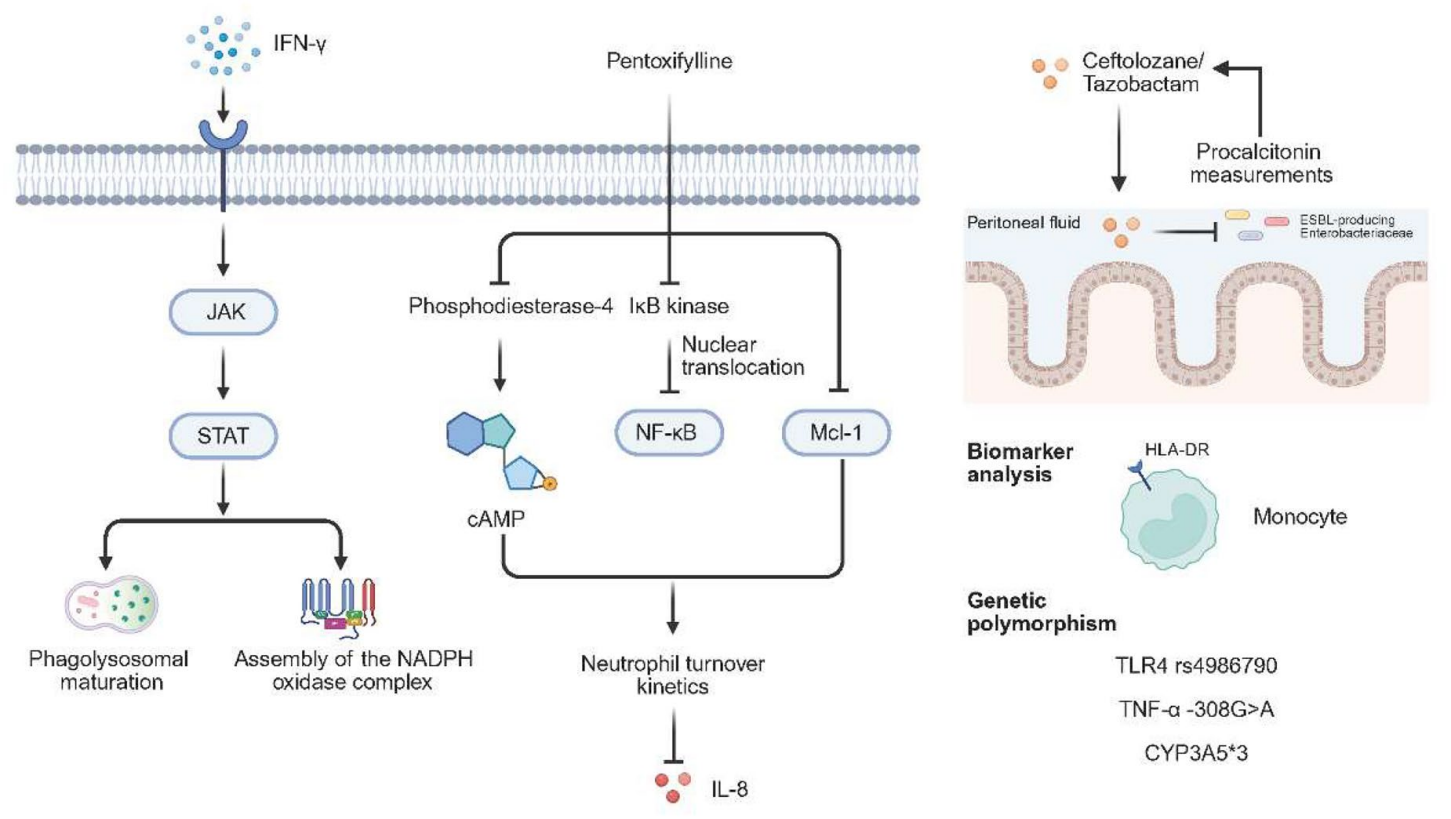
Immunomodulation and personalized anti-infective strategies in sepsis management. IFN-γ activates the JAK–STAT signaling pathway to promote phagolysosome maturation and stabilize NADPH oxidase complex assembly at the transcriptional level. Pentoxifylline exerts multimodal immunomodulatory effects, including inhibition of PDE4 to increase intracellular cAMP levels, inhibition of IKK activity to attenuate NF-κB nuclear translocation, and transcriptional downregulation of the anti-apoptotic protein Mcl-1, thereby reducing plasma IL-8 levels by approximately 48% and significantly mitigating progression to multiple organ dysfunction syndrome. Ceftolozane/tazobactam, a next-generation β-lactam/β-lactamase inhibitor combination, demonstrates superior penetration into peritoneal fluid and maintains efficacy against ESBL-producing Enterobacteriaceae in abdominal sepsis. Biomarker-guided strategies incorporating serial PCT measurements shorten antibiotic exposure by an average of 3.2 days. Flow cytometry-based surface marker analysis (e.g. HLA-DR expression on monocytes) enables personalized immunomodulation. Functional genetic polymorphisms, including pathogen recognition receptor TLR4 rs4986790, cytokine gene TNF-α − 308 G > A, and drug-metabolizing enzyme CYP3A5*3, are associated with sepsis susceptibility, patterns of organ failure, and pharmacokinetic responses, providing a foundation for algorithm-guided drug and dose selection. IFN-γ, interferon-gamma; JAK, Janus kinase; STAT, signal transducer and activator of transcription; NADPH, nicotinamide adenine dinucleotide phosphate; PDE4, phosphodiesterase-4; cAMP, cyclic adenosine monophosphate; IKK, IκB kinase; NF-κB, nuclear factor kappa-light-chain-enhancer of activated B cells; Mcl-1, myeloid cell leukemia-1; IL-8, interleukin-8; ESBL, extended-spectrum β-lactamase; PCT, procalcitonin; HLA-DR, human leukocyte antigen-DR; TLR4, toll-like receptor 4; TNF-α, tumour necrosis factor-alpha; CYP3A5, cytochrome P450 family 3 subfamily A member 5.

Contemporary immunomodulatory strategies targeting PMNs seek to achieve a delicate therapeutic equilibrium in sepsis management: enhancing their critical antimicrobial functions while restraining pathological inflammation that drives multiorgan injury (some therapeutic strategies targeting PMNs as shown in Table 3). Pioneering cytokine-directed approaches utilize recombinant interferon-γ (IFN-γ) to fundamentally reprogram PMNs functional capabilities [98]. Through JAK-STAT signalling pathway activation, IFN-γ transcriptionally upregulates phagolysosomal maturation machinery and stabilizes NADPH oxidase complex assembly (Figure 3). This molecular reprogramming is clinically relevant, as mechanistic and translational studies indicate that pharmacologic modulation of neutrophil metabolic and signalling pathways can enhance phagocytic and oxidative effector functions, providing a rationale for exploring such strategies to improve infection control in sepsis [26,99].

**Table 3.** Immunomodulatory strategies and mechanisms involving PMNs.

Intervention	Main immunological target/mechanism	Key immunological/clinical effects	Evidence type / disease context	References
Betanin (beetroot component)	Modulates neutrophil ROS production, DNA damage and apoptosis	In human neutrophils *in vitro*, 2–500 μM betanin reduced ROS generation in resting and PMA-stimulated cells, decreased DNA damage at 24 h and increased caspase-3 cleavage products.	*In vitro* study using human peripheral blood neutrophils	[98]
Neutrophil elastase in pancreatic ductal adenocarcinoma (PDAC)	Neutrophil elastase degrades tumour-cell E-cadherin and alters adhesion	In PDAC cell monolayers, exposure to neutrophils or purified elastase caused E-cadherin degradation, loss of cell–cell adhesion and increased migration; in 112 PDAC biopsies, higher neutrophil infiltration was associated with lower E-cadherin expression.	*In vitro* functional assays and tissue cohort analysis in human PDAC	[30]
Pathogen-induced ectosome release from human neutrophils	Ectocytosis of membrane-derived vesicles carrying antimicrobial and endosomal/oxidase components	Infection of human neutrophils with Mycobacterium tuberculosis H37Rv or other microbes, or PMA stimulation, induced ectosome release within minutes; ectosomes expressed CD35, phosphatidylserine, Rab5, Rab7 and gp91phox, and release was not linked to apoptosis.	*In vitro* infection/activation models using human neutrophils	[103]
CXCL12–CXCR4 signalling in polymicrobial sepsis	Chemokine axis regulating bone marrow neutrophil egress during sepsis	In a murine CLP sepsis model, bone marrow CXCL12 expression decreased and splenic CXCR4 increased; CXCL12 blockade reduced neutrophil egress, lowered blood and peritoneal neutrophil counts, increased peritoneal bacterial burden and reduced survival.	Experimental murine cecal ligation and puncture (CLP) sepsis model	[104]
PCT-guided discontinuation of antibiotics in ICU sepsis	Biomarker-guided timing of antibiotic cessation	Decision-tree modelling based on ICU sepsis trials estimated that a PCT-guided discontinuation algorithm reduces antibiotic days, length of stay, number of blood cultures and total hospital costs by about 9% per patient, with safety consistent with underlying RCTs.	Model-based economic evaluation informed by randomized sepsis trials	[105]
Personalized inherent randomness of the immune system (novel platform for personalized immunotherapies)	Repeated immune challenges used to quantify subject-specific patterns and amplitude of immune-response variability over time	In animal experiments, responses to the same immune trigger and immunomodulatory regimen varied markedly between subjects, whereas within-subject response patterns over repeated challenges were relatively stable and characteristic for each individual.	Preclinical experimental and methodological study on personalized immunomodulation	[106]

Complementing cytokine modulation, sophisticated apoptosis-targeting therapeutics address the biphasic dysregulation observed in sepsis—where accelerated apoptosis induces immunoparalysis while delayed apoptosis perpetuates tissue injury. Pharmacological agents like pentoxifylline (administered *via* 1.5 mg/kg/hr IV infusion) exert multimodal regulation through: Phosphodiesterase-4 inhibition that elevates intracellular cyclic AMP concentrations; Suppression of IκB kinase activity that attenuates NF-κB nuclear translocation; Transcriptional downregulation of anti-apoptotic Mcl-1 protein expression [100].

This tripartite mechanism restores physiological neutrophil turnover kinetics in clinical sepsis, reducing plasma IL-8 concentrations by 48% and significantly attenuating progression to multiple organ dysfunction syndrome (MODS) [33]. Parallel chemotaxis-optimizing approaches investigate CXCR2 allosteric modulators (e.g. reparixin) that enhance directional migration precision in microfluidic models while preventing destructive bystander tissue infiltration—though clinical validation remains ongoing.

Concurrently, transformative anti-infective paradigms have evolved beyond empirical regimens toward biomarker-directed precision therapy [89]. Critical temporal analyses reveal each hour’s delay in appropriate antibiotic administration increases mortality risk by 7.6% (adjusted OR 1.32, 95% CI 1.15–1.47), necessitating rapid diagnostic platforms like MALDI-TOF mass spectrometry for pathogen identification [50,101]. Novel antimicrobial agents expand therapeutic options against multidrug-resistant pathogens; ceftolozane/tazobactam, a next-generation β-lactam/β-lactamase inhibitor combination, demonstrates superior penetration into peritoneal fluid and maintains efficacy against extended-spectrum β-lactamase (ESBL)-producing Enterobacteriaceae in abdominal sepsis [29]. Biomarker-guided stewardship protocols incorporating serial procalcitonin (PCT) measurements reduce antibiotic exposure duration by 3.2 days on average while decreasing carbapenem-resistant Enterobacteriaceae colonization by 38% [30]. Adjunctive therapeutic synergies are increasingly explored, particularly combining antimicrobials with extracorporeal blood purification techniques that remove inflammatory mediators or immunomodulators that restore immune homeostasis [102].

The frontier of personalized sepsis management integrates multidimensional immunological phenotyping with pharmacogenomic insights. Comprehensive immune monitoring encompasses flow cytometric surface marker analysis (such as HLA-DR expression on monocytes), functional assays of phagocytic capacity, and transcriptomic profiling of leukocytes. These approaches together enable tailored immunomodulation. In practice, they can guide targeted immunosuppression using anti-TNF biologics or corticosteroids for hyperinflammatory phenotypes, and IFN-γ or GM-CSF therapy for immunoparalysis states [103]. Genetic stratification further refines precision medicine. Functionally significant polymorphisms in pathogen recognition receptors (TLR4 rs4986790), cytokine genes (TNF-α −308 G > A), and drug-metabolizing enzymes (CYP3A5*3) correlate with sepsis susceptibility, organ failure patterns, and pharmacokinetic responses. These associations enable algorithm-guided drug and dose selection [104]. Preclinical validation remains indispensable, with humanized mouse models and *ex vivo* whole-blood stimulation systems providing critical platforms for evaluating patient-specific therapeutic regimens before clinical implementation [105,106].

#### 3.3.3. Therapeutic balancing of immunosuppression and immunostimulation in sepsis

Although the need to dynamically balance hyperinflammation and immunosuppression has been recognized for more than three decades, progress towards implementing temporally precise, immunity-tailored therapies has been limited, and robust tools to guide such interventions in sepsis are still lacking [106,107]. During the initial hyperinflammatory phase (typically 0–72 h post-infection), systemic inflammatory response syndrome (SIRS) manifests through cytokine storms (TNF-α > 500 pg/mL, IL-6 > 1000 pg/mL), complement cascade overactivation (C5a >40 ng/mL), and neutrophil extracellular trap (NET)-mediated cytotoxicity [107,108]. At this juncture, targeted immunosuppression, employing TNF-α antagonists (infliximab 5 mg/kg), low-dose corticosteroids (hydrocortisone 200 mg/day), or extracorporeal CytoSorb^®^ adsorption, can attenuate mitochondrial permeability transition pore (mPTP) opening and capillary leakage. However, excessive suppression risks precipitating compensatory anti-inflammatory response syndrome (CARS), characterized by lymphocyte apoptosis (CD4+ T-cell depletion >80%), monocyte deactivation (mHLA-DR <8000 antibodies/cell), and impaired bacterial clearance [108].

Paradoxically, the subsequent immunosuppressive phase (days 4–14) features profound immunoparalysis: antigen-presenting cell dysfunction (MHC-II internalization), T-cell exhaustion (PD-1 expression >90%), and endotoxin tolerance (LPS-induced TNF-α < 200 pg/mL). This necessitates strategic immunostimulation *via* recombinant IFN-γ (100 μg/m^2^ thrice weekly), GM-CSF (250 μg/m^2^/day), or PD-1/PD-L1 checkpoint inhibitors to restore microbial clearance. Current monitoring integrates several parameters. These include flow cytometric mHLA-DR quantification (paralysis threshold <15,000 AB/cell), lymphocyte subset enumeration (CD4+/CD8+ ratio <1.0), and transcriptomic signatures such as TREM-1/PD-L1 co-expression. Notwithstanding these advances, personalized immunobalancing remains constrained by kinetic phenotype switching, driving research toward multi-omics integration (single-cell RNA-seq, serum proteomics) with machine learning predictive algorithms [109,110].

Within this immunological continuum, polymorphonuclear neutrophils (PMNs) exhibit context-dependent duality. Their protective armamentarium encompasses several mechanisms. First, FcγRI (CD64)-mediated opsonophagocytosis is enhanced 4.7-fold during Pseudomonas aeruginosa bacteremia through PI3K-dependent actin polymerization. Second, precision degranulation results in targeted release of α-defensins (HNP1–3) and cathelicidin (LL-37), achieving 99.3% bacterial killing at infection foci. Third, controlled NETosis extrudes chromatin to capture pathogens while preserving vascular integrity through DNase1-mediated clearance [26,81,101]. Conversely, dysregulated PMNs activation unleashes pathogenic cascades. Reactive oxygen species (ROS) overproduction (>50 μM H_2_O_2_ equivalents) induces endothelial junctional protein degradation *via* VE-cadherin cleavage. Proteolytic enzyme release (matrix metalloproteinase-9 and elastase) mediates alveolar–capillary barrier disruption, reflected by bronchoalveolar lavage protein levels >500 μg/mL. In addition, unresolved NET formation provokes microthrombosis through histone H4–mediated platelet activation (80% increase in P-selectin expression) and induces ferroptosis in renal tubular epithelium [84,111].

This functional paradox necessitates therapeutic strategies that preserve PMNs antimicrobial efficacy, such as CXCR2 antagonists (reparixin) optimizing chemotactic precision, while constraining bystander injury through PAD4 inhibitors (GSK484) blocking pathological NETosis [26]. The ultimate challenge remains real-time modulation of PMNs functional phenotypes aligned with the patient’s evolving immunodynamic status, requiring advanced biosensor platforms for granular immune monitoring [112].

## 4. Conclusion

Emerging research trends in sepsis include the use of machine learning and artificial intelligence. In a study on predicting preoperative and postoperative coagulopathy in patients with trauma, 10 machine learning models were developed to predict traumatic coagulopathy (PPTIC) based on preoperative indicators. The random forest model was the most effective, achieving an area under the receiver operating characteristic of 0.91 in external validation. This approach can help clinicians identify high-risk patients and implement timely interventions, which may also be applicable to sepsis patients considering the association between coagulopathy and sepsis [73].

Another trend is the investigation of new biomarkers. For example, heat shock protein 27 (HSP27) has been studied as a biomarker for sepsis. In a retrospective cohort study of septic ICU patients, median serum HSP27 levels in septic patients were significantly higher than those in non-septic ICU controls and healthy controls. Non-survivors had significantly higher median HSP27 levels compared to survivors, and multivariate logistic regression analysis confirmed the association between HSP27 levels and 28-day mortality in sepsis patients. ROC curve analysis showed an area under the curve (AUC) of 0.720 for HSP27 in predicting sepsis prognosis, suggesting its potential as a biomarker [74]. Additionally, the role of necroptosis in sepsis is being explored. A bibliometric analysis showed that an increasing number of studies have shown that necroptosis plays an important role in inflammatory diseases, including sepsis, and inhibitors of necroptosis may have great potential in the treatment of sepsis [113].

The future prospects for immune model development in sepsis are promising. There is a focus on developing more accurate and physiologically relevant models. For example, constructing a prognostic model based on immune-related genes (IRGs) has shown potential. In one study, a prognostic model based on 22 differentially expressed IRGs was developed to predict 28-day mortality in sepsis. The model had a high accuracy, with an AUC value of 0.879 in the discovery cohort, and was also validated in external datasets. The higher risk score was positively associated with 28-day mortality and the development of immunosuppression, providing a new tool for personalized treatment strategies [114].

Another aspect is the use of immune models to understand the complex immune cell interactions in sepsis. A comprehensive analysis of immune features in sepsis identified two sepsis immune subtypes based on differential immune cell infiltration. Weighted gene co-expression network analysis was used to identify key module genes, and a diagnostic model was constructed based on 11 immune-related genes. This type of analysis can provide a better understanding of the immune characteristics of sepsis and potentially lead to the development of more targeted immunotherapies [115]. Additionally, understanding the role of programmed cell death in sepsis through immune models is an emerging area. Immunoadjuvant therapies designed to modulate the various types of programmed cell death, such as apoptosis, autophagy, NETosis, pyroptosis, ferroptosis, and necroptosis, which contribute to immunosuppression in sepsis, are being investigated, and immune models will be crucial in evaluating the effectiveness of these therapies.

## Data Availability

There is no data associated with this research.
